# Supplementary material for: Splicing activates transcription from weak promoters upstream of alternative exons
Source: Nat Commun. 2023 Jun 10;14:3435. doi: 10.1038/s41467-023-39200-2 (PMC10256964; doi:10.1038/s41467-023-39200-2)
Supplement: Supplementary file 1 — Supplementary information [file 41467_2023_39200_MOESM1_ESM.pdf]

Supplementary Information

Splicing activates transcription from weak promoters upstream of alternative exons

Maritere Uriostegui-Arcos[1], Steven T. Mick[1], Zhuo Shi[2], Rufuto Rahman[1], Ana Fiszbein[1*]

[1] Biology Department, Boston University, Boston USA 02215
[2] Biology Department, Massachusetts Institute of Technology, Cambridge USA 02139

*Corresponding author. Email: anafisz@bu.edu

Supplemental figures

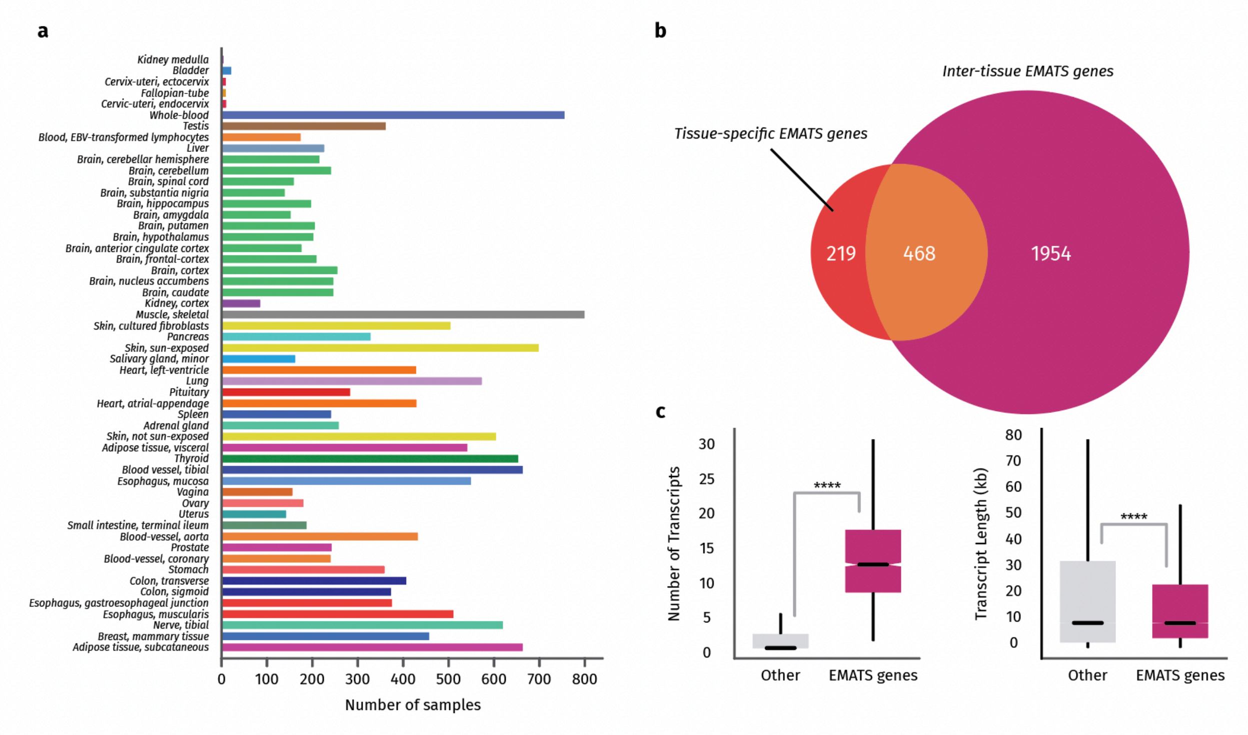

Supplementary Figure 1.

(a), distribution of samples per sub-tissue in GTEx. (b), overlap between human EMATS genes identified across all tissues and samples from GTEx (Inter-tissue EMATS genes) and those identified per tissue (Tissue-specific EMATS genes). (c), distribution of total transcript length for isoforms of genes with EMATS structure and other genes. (d), distribution of the number of isoforms per gene for genes with EMATS structure and other genes. Statistical significance is indicated by asterisks (****p < 0.0001; two-sided independent t-test); in b and c, n (other genes) = 55,864, and n (EMATS) = 2,306.

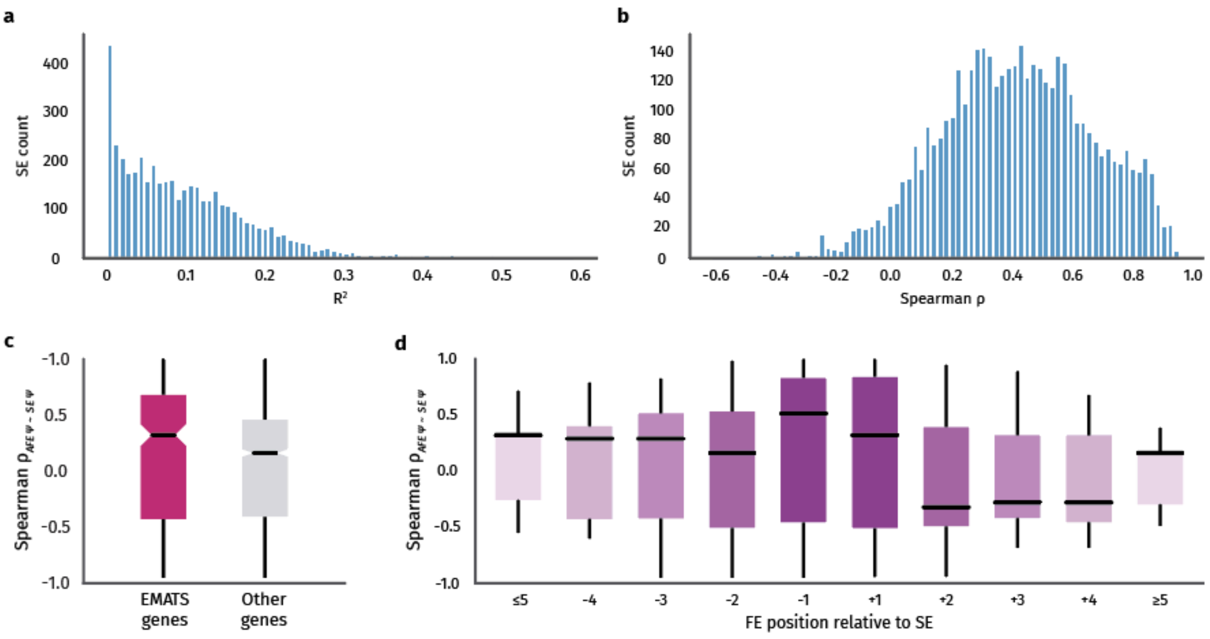

Supplementary Figure 2

(a,b) distribution of r^2 values (a) and Spearman rho values **(b)** for EMATS genes with at least 70 paired skipped exon-gene expression observations. **(c, d)** distribution of the spearman correlation between changes in AFE PSI and changes in SE PSI during SARS-CoV-2 infection (n = 3 mock NHBE cell samples and n = 3 SARS-CoV-2 infected NHBE cell samples) for non-EMATS genes compared to EMATS genes **(c)** binned by the position of the promoter relative to the position of the SE located in an EMATS structured **(d).** Promoters are numbered with increasing positive values downstream of the SE and with decreasing negative values upstream of the SE.,

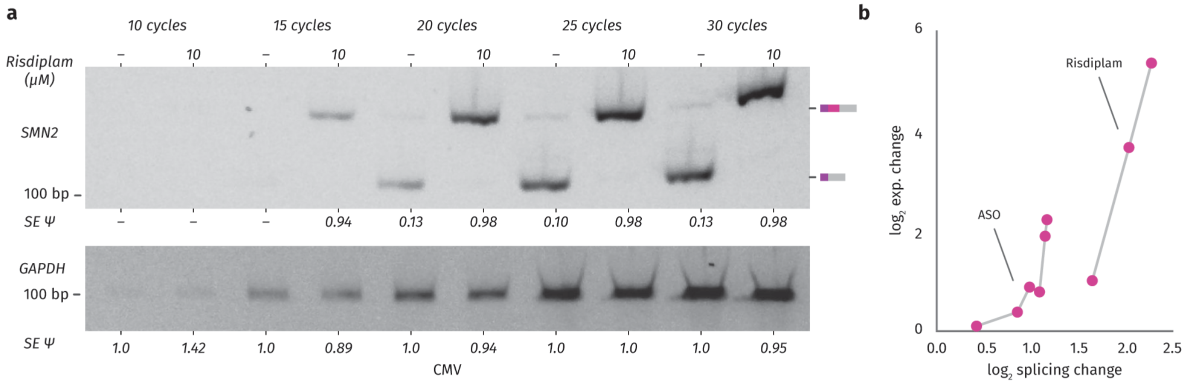

Supplementary Figure 3

a. HEK293T-A2 stable cell lines expressing SMN2 splicing reporter were treated with Ethanol (-) or 0.1 μM, 1.0 μM, and 10 μM of Risdiplam for 24 h. Inclusion of alternative exon 7 in SMN2 was evaluated by RT-PCR with different cycles. Quantification of densitometry analyses was carried out with Fiji, +Exon/-Exon ratios are shown at the bottom of each lane, and GAPDH is shown as a control. n=3 experiments, with up to 3 biological replicates and 3 technical replicates. **B.** change in expression compared to change in splicing for cells expression SMN2 reporter following treatment with Risdiplam or a specific ASO for 24 h.

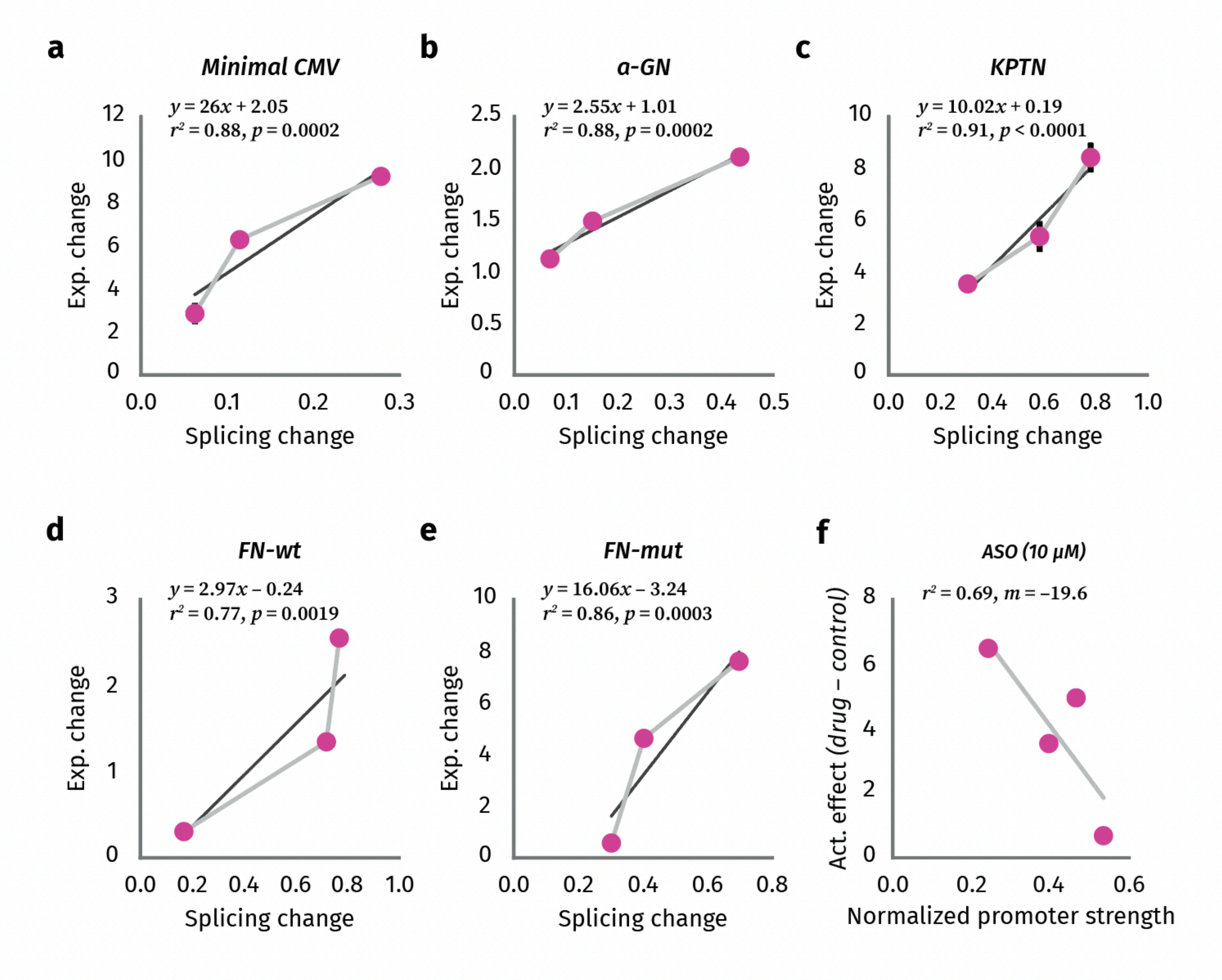

Supplementary Figure 4

(**a-e**) linear regression of splicing changes and gene expression after Risdiplam treatment with three different concentrations driven by a minimal CMV promoter (**a**), an alpha-globin (a-GN) promoter (**b**), a KTPN promoter (**c**), the fibronectin wilt type (FN-Wt) promoter (**d**), and the fibronectin mutated (FN-Mut) promoter (**e**). (**f**) linear regression of gene expression changes in SMN2 splicing reporter following ASO treatment.

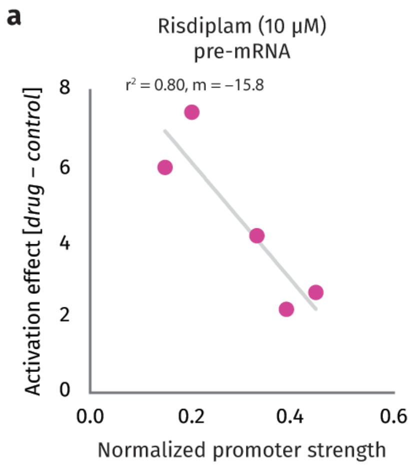

Supplementary Figure 5

Linear regression of gene expression changes in SMN2 splicing reporter following Risdiplam treatment in newly synthesized RNA for stable lines under the regulation of the four different human promoters tested and promoter strength measured by basal RT-qPCR in control conditions.

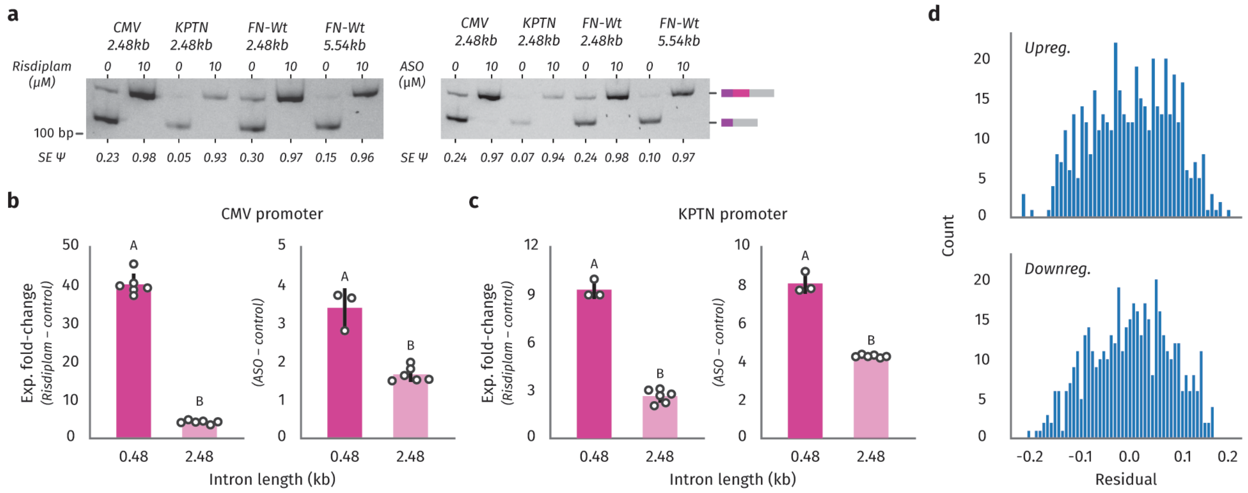

Supplementary Figure 6

a. HEK293T-A2 stable cell lines expressing SMN2 splicing reporter were treated with Risdiplam or the ASO for 24 h. Inclusion of alternative exon 7 in the SMN2 reporter was evaluated by RT-PCR and quantification of densitometry analyses was carried out with Fiji, and the +Exon/-Exon ratios are shown at the bottom of each lane. This is a representative image from three independent results. **b,c.** HEK293T-A2 stable cell lines expressing SMN2 splicing reporter under the regulation of CMV (b) or KPTN (c) promoter and two different intronic sequences were treated with Risdiplam (left) or a scramble antisense oligonucleotide (-) or a specific ASO modeled after the Spinraza (+) for 24 h (right). Expression of the SMN2 reporter was evaluated by RT-qPCR. The scatter plot with bars represents the mean ± standard error, and individual dots represent individual data points; n=3 experiments, with up to 3 biological replicates and 3 technical replicates. Bars with the same letter indicate no significant difference between means, as determined by one-way ANOVA (Tukey's HSD test, $p < 0.0001$) comparing across groups. **d.** the distribution of residuals for the fraction of differentially expressed genes regressed on the fraction of differentially skipped exons, stratified by upregulation and downregulation.

Supplemental tables

Supplementary table 1

Oligonucleotides used in this study.

Oligonucleotides	
SMN2_genomic_F	TAACTGCAGCCTAATAATTG
SMN2_genomic_R	ACAACCAACCAGTTAAGTATG
SMN2_for_Gibson-assembly_F	ACAGGCATTGGAACTTCAGCATCGATGTAACTGCAGCCTAATAATTG
SMN2_for_Gibson-assembly_R	CCTCCCCAGCTGTGCCCCCCTCGAGACAACCAACCAGTTAAGTATG
SMN2_E5_endogenous-F	CCACCACCTCCCATATGTC
SMN2_E8_ endogenous -R	AGCCATGTCCACCAGTTAG
SMN2_total_endogenous_F	GCTCACATTCCTTAAATTAAGGAGAAA
SMN2_total_endogenous_R	TCCAGATCTGTCTGATCGTTTCTT

Minimal_CMV	TACGAAGTTATATGGATCCATATGGGTAGGCGTGTACGGTGGG AGGTCTATATAAGCAGAGCTCTAGCGTTTAAACTTAAGCTTCCA
a-GN_promoter_F	TACGAAGTTATATGGATCCATATGTAGTGACTATCGCCAGAGG
a-GN_promoter_R	TGGAAGCTTAAGTTTAAACGCTAGGCGCCAGGGTTTATGCTTG
KPTN_promoter_F	TACGAAGTTATATGGATCCATATGACACAGCATACAGCGAGAG GC
KPTN_promoter_R	TGGAAGCTTAAGTTTAAACGCTAGGGCTGATGACGTACGGAAG CT
FN_Wt_promoter_F	TACGAAGTTATATGGATCCATATGATGACCGCAAAGGAAACCG
FN_Wt_promoter_R	TGGAAGCTTAAGTTTAAACGCTAGAAGTTGTGGCTGCAGGTC
FN_Mut_promoter	TACGAAGTTATATGGATCCATATGATGACCGCAAAGGAAACCG AAAAAAAGTTGTCTTGCCCCAGTCCTGGCGGGCCATCAGCATC TCTTTTGTTCGCTGCGAACCCACAGTCCCCCGTGGCTTCACCC GGAGCCCGGGCCCCTCGGCGCGCGGTCGGCTGCGGCGGCCG GCGGGCGGGCGGGCGGGTGGGGTGGGGCGGGGCGGGGAC AGCCCGGCGGGTCTCTCCTCCCCCGCGCCCCGGGCCTCCAGA GGGGCGGGAGGGGACCGTCCCATATAAGCCCCGGCTCCCGG CGCTCGGACGCCCGCGCCGGCTGTGCTGCACAGGGGGAGGA GAGGGAACCCCAGGCGCGAGCGGGAAGAGGGGACCTGCAGC CACAACTTCTAGCGTTTAAACTTAAGCTTCCA
Total-ss-F	AGCTGGACGGCGACGTAAAC
Total-ss-R	TGCCGGTGGTGCAGATGAAC
Intron_2kb_F	GGGAAACAAGCACAGTCTTCAC
Intron_2kb_R	ATCTGCCAGCCTTGGCTATATG
Intron 2kb for Gibson assembly F	AGGCATTGGAACTTCAGCATGGGAAACAAGCACAGTCTTCAC
Intron 2kb for Gibson assembly R	TTATTAGGCTGCAGTTACATATCTGCCAGCCTTGGCTATATG
Intron_5kb_F	GGGATTGGCAGGCTATCTC
Intron_5kb_R	GCTACCCAGAAGGCTAAGG
Intron 5kb for Gibson assembly F	AGGCATTGGAACTTCAGCATGGGATTGGCAGGCTATCTC
Intron 5kb for Gibson assembly R	TTATTAGGCTGCAGTTACATGCTACCCAGAAGGCTAAGG
Inclusion_Exclusion_E7_F	CAAAGTGGAGGACCCAGTACC

Inclusion_Exclusion_E7_R	GCGCATGAACTCCTTGATGAC
SMN2_total_F	GCTTCAAGGTGCGCATGGAG
SMN2_total_R	GCCCTTGGTCACCTTCAGCTTC
SMN2_nascent_(intron)_F	CCTACTGACCCTGTCCTTATTG
Promoter_Cut&Run_F	TGGAAGCTTAAGTTTAAACGCTAG
CMV_promoter_Cut&Run_R	TGACGCAAATGGGCGGTAG
KPTN_promoter_Cut&Run_R	GCAGCTTCCGTACGTCATCAG
Gen_body_Cut&Run_F	GGAAGGTGCTCACATTCC
Gen_body_Cut&Run_F	CAACTTTCTAACATCTGAAC
GAPDH_	GTTCCAATATGATTCCACCC
GAPDH_R	CTTGATTTTGGAGGGATCTCG
GAPDH_nascent_F	AATCCCATCACCATCTTCCAG
GAPDH_nascent_R	GAGCCACACCATCCTAGTTG
P1_Exon_1_F	CAAAGTGGAGGACCCAGTACC
P1_Intron_1_R	TCAGTCTGCCCATGGCAAG
P2_Intron_1_F	CAAAAAGAAGGAAGGTGCTCACATT
P2_Exon2_R	CAACTTTCTAACATCTGAAC
P3_Intron_2_F	CAGTGCCACGGTTGTCCATTC
P3_Exon_3_R	TTCCTCCTCGTCCTCCTGATCC
