# Supplementary material for: Splicing activates transcription from weak promoters upstream of alternative exons
Source: Nat Commun. 2023 Jun 10;14:3435. doi: 10.1038/s41467-023-39200-2 (PMC10256964; doi:10.1038/s41467-023-39200-2)
Supplement: Supplementary file 3 — Description of Additional Supplementary Files [file 41467_2023_39200_MOESM3_ESM.pdf]

## Description of Additional Supplementary Files

File Name: Supplementary Data 1
Description: A list of EMATS genes and pertinent exon information: column 1 is the gene's Ensembl gene identifier; column 2 the gene symbol; column 3 the first exon (on a 0-based, half-open interval) in generic, genome-browser format; column 4 the skipped exon in an identical format; column 5 the kilo-base distance between the two exons' 5' coordinates, where the positive values indicate the skipped-exon's downstream orientation relative to the first exon; and column 6 the gene's strand, where plus is forward and minus is reverse.

File Name: Supplementary Data 2
Description: A matrix of tissue-specific EMATS genes, where column 1 is the gene symbol and the remaining columns the main tissue sites (i.e., tissues without sub-type stratification) represented in GTEx. 1 at a particular row, column index indicates the row's gene is EMATS-specific to the column's tissue.
